# Proposed approaches for public health surveillance: A literature review for the Canadian National HIV Surveillance Program

**DOI:** 10.14745/ccdr.v51i05a06

**Published:** 2025-05-01

**Authors:** Anita Robert, Wes Martin, Leigh Jonah, Dana Paquette, Joseph Cox, Laura H Thompson

**Affiliations:** 1 Centre for Communicable Disease and Infection Control, Public Health Agency of Canada, Ottawa, ON; 2 Centre for Surveillance and Applied Research, Public Health Agency of Canada, Ottawa, ON; 3 School of Population and Global Health, Department of Global and Public Health, McGill University, Montréal, QC

**Keywords:** HIV, surveillance, diagnosis, review literature as topic, public health practice

## Abstract

**Background:**

The National HIV Surveillance Program, managed by the Public Health Agency of Canada, is a passive surveillance system that collects de-identified data on HIV cases in Canada. Regular review of this surveillance system is required to maintain its accuracy, effectiveness and relevance in the face of a changing HIV epidemic. The National HIV Surveillance Program is undergoing a comprehensive review and renewal process with the aim of identifying and implementing potential improvements to meet the information needs of communities, service providers, researchers, provinces and territories and the federal government more effectively.

**Methods:**

A non-systematic literature review was conducted in June to July 2023, with 3,521 articles found and 105 included.

**Objective:**

This literature review aimed to identify proposed approaches for public health surveillance, with an emphasis on HIV surveillance and identify key findings relating to the following themes: surveillance system infrastructure, data collection, ethical considerations and stakeholder relationships.

**Results:**

Key findings from the literature review pertained to standardization and centralization of data collection; collection of demographics, disease staging, social determinants of health and other data elements; and linking surveillance systems to other data sources or other surveillance systems. Additional findings concerned legislative and policy review, privacy strategies, informed consent, ethical surveillance system design, stakeholder consultation at all stages, knowledge translation and ensuring adequate resourcing.

**Conclusion:**

In future work, lessons resulting from the literature review will be combined with evidence from other components of the overall review of Canada’s HIV surveillance system. Together, this information will be further assessed and prioritized for possible implementation after consultation with data providers and communities.

## Introduction

In 2022, there were 1,833 newly diagnosed HIV cases in Canada ( ( ( ((1))))). Given the persistence of HIV and other sexually transmitted and blood-borne infections (STBBIs) in Canada, the federal government highlighted surveillance systems as a key evidence source to inform programs and policies in its goal to reduce the burden of STBBIs. It committed to strengthening these systems in the Government of Canada’s STBBI Action Plan (2024–2030) ( ( ( ((2,3))))). Therefore, high-quality HIV surveillance data remains critical to Canada’s STBBI response.

Public health surveillance systems should be updated as proposed surveillance approaches, or the epidemiology of the condition of interest evolves. The National HIV Surveillance Program compiles data on HIV diagnoses in Canada from various sources: line-listed datasets and case report forms on individual HIV diagnoses reported in the provinces and territories (PT), aggregate data tables on perinatal exposure to HIV from the Canadian Perinatal HIV Surveillance Program, aggregate data tables on positive HIV tests during immigration medical exams provided by Immigration, Refugees and Citizenship Canada (IRCC) and HIV-related mortality data from Statistics Canada. In response to annual data requests sent to the PTs, line-listed datasets and case report forms with data on HIV diagnoses are received, data is cleaned and reformatted to match national HIV surveillance variable formats, PT-specific data is validated, national surveillance data is compiled and reports and infographics are prepared. Additional information on the operation of the surveillance system has been described elsewhere ( ( ( ((4))))). The review and renewal process initiated in 2021 represents the first comprehensive review of the National HIV Surveillance Program. This review and renewal process was undertaken to improve the surveillance system for harmonization of methods related to data reporting and collection across jurisdictions and to better meet community, program and policy engagement and evidence needs. As the epidemiology of HIV has evolved over time, the review and renewal ensure that the National HIV Surveillance Program remains relevant and allows for better alignment of the surveillance system with the goals of the Government of Canada’s STBBI Action Plan (2024–2030) ( ( ( ((2,3))))). The National HIV Surveillance Program is undergoing a review and renewal process to identify areas for improvement and implement needed changes to provide better quality data to data users. Along with technical assessments and stakeholder and other key informant consultations, a literature review was undertaken to identify proposed approaches in public health surveillance, emphasizing HIV surveillance systems, to advance HIV surveillance in Canada. This literature review was conducted as an information gathering exercise to identify proposed approaches implemented by other surveillance systems that will be discussed with data providers and community stakeholders for potential implementation during the renewal process. This article reports the findings of the review grouped across the following themes: surveillance system structure and methodology, data collection, ethical considerations and stakeholder consultation.

## Methods

A general, non-systematic literature review was performed in June to July 2023 to examine proposed approaches in local, provincial and national public health surveillance systems within Canada and globally. Follow-up for newly diagnosed HIV cases in Canada is completed locally, with required reporting to provincial/territorial health authorities and subsequent reporting to the national HIV surveillance program. The National HIV Surveillance Program reports on national trends in new HIV diagnoses stratified by various epidemiological factors. The following topics, determined to be relevant to the National HIV Surveillance Program, were examined: surveillance system processes and methodologies; data reporting methods; data infrastructure; considerations for collection, analysis and use of sociodemographic and epidemiological data; community engagement; ethical considerations; inter-surveillance system linkage; and program and policy engagement.

Distinct search strategies were developed for PubMed, Embase and Google Scholar to address each topic. PubMed and Embase were selected, as their combined use results in a high coverage rate of publications for research ( ( ( ((5))))) and this search was supplemented by Google Scholar due to the large volume of publications included ( ( ( ((6))))). Various search terms were developed for each aspect of the given topic. Search terms used for “surveillance” included: surveillance, monitoring, disease surveillance, passive surveillance, etc. The topics were divided equally across two reviewers responsible for search strategy development, database searches, article screening and assessment for inclusion, article detail extraction and analysis. Given that this was a non-systematic literature review and given the volume of articles identified for screening, each article was only reviewed by one of the two reviewers and no procedure existed for discordant assessments or disagreements between reviewers. Data extraction was completed by entering data into structured evidence tables on citation information, inclusion/exclusion decision, rationale for decision regarding inclusion, notes and the main message for the National HIV Surveillance Program.

**Figure 1:**
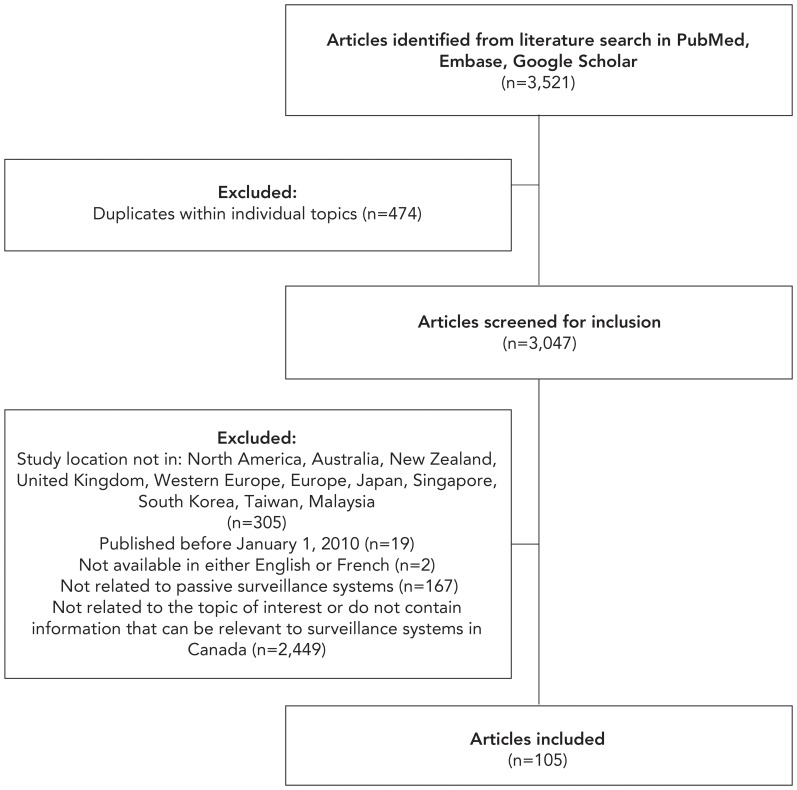
Flowchart of Article Screening in Literature Review^a^ ^a^ As literature searches were conducted separately for each topic, the number of duplicates only includes articles identified as duplicated within a particular topic. As articles could be included in multiple themes, the number of duplicates does not include articles appearing under multiple themes

Overall, 3,521 articles were identified for screening (**Figure 1**). Articles were assessed for inclusion based on criteria that included relevance, recency and geographic location. By including articles published on or after January 1, 2010, the authors were able to obtain information on improvements to public health surveillance in response to various events over time (i.e., swine flu pandemic, Ebola, COVID-19 pandemic, etc.) and including articles by study location allowed for the extraction of relevant information from jurisdictions with similar public health contexts. Although this literature review was completed as part of the review of the National HIV Surveillance Program, articles related to other conditions were included, as key findings from these articles can also be applied to HIV surveillance. Occasionally, articles with geographic locations outside of the listed regions or published before 2010 were included if reviewers deemed them particularly relevant for Canadian HIV surveillance. Included articles not meeting one or more inclusion criteria are detailed in the **Appendix, ****Table A1**. Such articles held lessons for the renewal of the National HIV Surveillance Program and met the other relevant criteria for inclusion: contains information that can be relevant to surveillance systems in Canada, related to passive surveillance systems, contains information that is otherwise relevant to surveillance system modernization and available in either English or French. Articles were not restricted by study design. While there was no specific separate search for grey literature, the intention in the use of Google Scholar as a searched database was to capture relevant grey literature Thus, 3,416 articles were excluded across all topics.

xPractices were deemed to be “proposed approaches” if their implementation could improve data collection and surveillance system methodology and infrastructure, ensure ethical public health surveillance and improve stakeholder engagement for the National HIV Surveillance Program. Proposed approaches are potential actions for the National HIV Surveillance Program supported by the literature. Although each proposed approach is evidence-based, these have not been vetted for risk or bias. Practices and processes from included articles were grouped into key findings for the National HIV Surveillance Program’s renewal. Individual articles could support multiple findings. Thematic analysis was performed using an open-coding system to derive themes through an inductive process, where the main message or key finding for the National HIV Surveillance Program from each included article was derived. Individual main messages were inductively grouped based on similarity to each other into key findings. Key findings were grouped into the four larger themes and subthemes based on surveillance system development, operation and evaluation. The process was conducted manually and no formal qualitative analysis procedure or software was used. The results of this review are separated into “Novel findings,” representing potentially new proposed approaches and processes for the National HIV Surveillance Program and “Long standing public health practices,” representing practices or processes that have been consistently used in public health surveillance. “Novel findings” were related to modernization and updating surveillance systems and were similar to findings identified from previously completed steps of this review process. “Long standing public health practices” were most often practices for ongoing surveillance system operation and were already in place at other surveillance systems both in Canada and in other jurisdictions.

## Results

### Key findings

Literature searches were conducted separately for each topic and 3,521 articles were identified across all sources. Article exclusions for duplicates and other reasons are outlined in Figure 1. Overall, 105 articles were included. One third (n=33) were published during or post-COVID-19 pandemic (2020 onwards). The geographic distribution of articles shows that half came from North America (n=52) and 17.1% (n=18) compiled information about multiple countries. Key findings were derived using an inductive approach, where articles included were analyzed to identify any practices or processes potentially applicable to the National HIV Surveillance Program. Post-literature review, the findings were grouped into four key themes: surveillance system structure and methodology, data collection, ethical considerations and stakeholder engagement.

### Novel findings

Novel findings were identified related to surveillance system structure and methodology, data collection, ethical considerations and stakeholder consultation. (**Table 1**). Most of the findings were supported by multiple articles, with additional details found in Table 1.

**Table 1:** Novel findings by theme and surveillance system stage

Surveillance system stage where relevant	Novel findings	Articles that support findings	Examples of novel findings
**Surveillance system structure and methodology**			
Surveillance system development	1. Enhance timeliness of data with real-time or close to real-time surveillance for key metrics and data linkage between different surveillance systems (i.e., within the same level, or local surveillance systems to the national surveillance system) to ensure relevance of reporting and ability to respond quickly to transmission clusters (n=8).	Beyrer (2021) ( ( ( ((7))))), Ghosh (2012) ( ( ( ((8))))), Mao (2010) ( ( ( ((9))))), McGill (2023) ( ( ( ((10))))), Moore (2012) ( ( ( ((11))))), Morse (2012) ( ( ( ((12))))), Price (2021) ( ( ( ((13))))), Sullivan (2022) ( ( ( ((14)))))	Mao (2010) ( ( ( ((9))))): Description of the creation of the Chinese national, web-based HIV/AIDS information system. This system provides real-time data on HIV testing, prevention and treatment from provinces. Price (2021) ( ( ( ((13))))): This article discusses the construction of local surveillance systems in the United Kingdom for COVID by linking data from laboratory information management systems with electronic medical records. Data about specimens collected from laboratory information management systems is linked to information about patient admissions and discharges from electronic medical records. This system allows for the real-time monitoring of hospital onset COVID infections.
Surveillance system operation	2. Use web-based application to assist with data collection (n=3).	Cohen (2014) ( ( ( ((15))))), Hood (2017) ( ( ( ((16))))), Mao (2010) ( ( ( ((9)))))	Cohen (2014) ( ( ( ((15))))): Review of the status of the United States (US) National HIV Surveillance System in 2013. This surveillance system uses specialized web application developed by the US Centers for Disease Control and Prevention (CDC) to receive monthly de-identified data submissions from HIV surveillance programs. Hood (2017) ( ( ( ((16))))): A study using HIV epidemiological data and field service data from King County, Washington to assess the impact of misclassification of prior diagnoses on new diagnoses and partners services on linkage to care. Cases in the Laboratory Tracking Database, a database containing electronic laboratory data submissions, are matched to those in the Enhanced HIV/AIDS Reporting System (eHARS), a web-based HIV surveillance data repository, prior to submission.
**Data collection**			
Surveillance system development	3. Supplemental information obtained through passive surveillance systems with multiple internal or external data sources aside from other existing surveillance systems such as: laboratory data, survey data, clinical data or electronic health records, healthcare system data, testing data and population data (n=28).	Brandwagt (2019) ( ( ( ((17))))), Buchacz (2015) (18), Chapman (2011) ( ( ( ((19))))), Cohen (2014) ( ( ( ((15))))), Ghosh (2012) ( ( ( ((8))))), Goller (2010) ( ( ( ((20))))), Hall (2021) ( ( ( ((21))))), Hood (2017) ( ( ( ((16))))), Jeong (2022) ( ( ( ((22))))), Jones (2011) ( ( ( ((23))))), Jung (2018) ( ( ( ((24))))), Kliewer (2010) (25), Kraut (2023) (26), Leal (2010) ( ( ( ((27))))), Moore (2012) ( ( ( ((11))))), Morse (2012) ( ( ( ((12))))), Newton (2012) ( ( ( ((28))))), Paulukonis (2014) ( ( ( ((29))))), Pierce (2017) ( ( ( ((30))))), Price (2021) ( ( ( ((13))))), Schmidt (2019) ( ( ( ((31))))), Shaw (2011) ( ( ( ((32))))), Sullivan (2022) ( ( ( ((14))))), Tanaka (2021) ( ( ( ((33))))), Weston (2018) ( ( ( ((34))))), WHO (2021) ( ( ( ((35))))), Wijayasri (2016) (36), Willis (2019) ( ( ( ((37)))))	Brandwagt (2019) ( ( ( ((17))))): Discusses the evaluation of the national invasive meningococcal disease surveillance system in the Netherlands, created by linking data from clinical datasets, laboratory datasets and serogroup/subtype data from the national reference laboratory. Data from clinical and laboratory sources are merged with data on serotype and subtype information obtained from further testing at the national reference laboratory. Hall (2021) ( ( ( ((21))))): This article discusses the creation of the HOSTED (Household Transmission Evaluation Database) surveillance system for household COVID19 transmission, developed by merging data from the Second Generation Surveillance System at Public Health England, National Health Service Personal Demographics Service data. The National Health Service Personal Demographics Service dataset has information on the home addresses of people registered with a general physician; unique households are indexed using a Unique Property Reference Number. Data linkage with the Second Generation Surveillance System for COVID occurs in a secure data access environment, generating a pseudonymized dataset for analysis.
	4. Include: race and/or ethnicity data, CD4 count and/or HIV staging, gender, sexual orientation data, residence data, socioeconomic data elements (i.e., income, poverty level, educational), data elements related to migration (i.e., country of infection or country of origin, immigration) and additional variables (i.e., insurance, coverage, incarceration status, occupation, homeless, pre-exposure prophylaxis [PrEP], reason for test) (n=12).	Dickson (2015) (38), Ford (2012) ( ( ( ((39))))), Newton (2012) ( ( ( ((28))))), Rice (2017) ( ( ( ((40))))), Rossi (2017) ( ( ( ((41))))), Schmidt (2019) ( ( ( ((31))))), Sullivan (2022) ( ( ( ((14))))), Cohen (2014) ( ( ( ((15))))), Croxford (2022) ( ( ( ((42))))), Beltran (2011) ((43)), Beyrer (2021) ( ( ( ((7))))), WHO (2021) ( ( ( ((35)))))	Dickson (2015) ( ( ( ((38))))): Overview of the epidemiology of HIV and AIDS in Aotearoa New Zealand since the inception of the surveillance system. This surveillance system collects data on age at diagnosis, gender, ethnicity, usual residence, likely means of infection, place of infection and initial CD4 cell count. Ford (2012) ( ( ( ((39))))): Identifies critical data and indicators related to HIV care and access to services, looks at the impact of the US National HIV/AIDS Strategy and looks at the data systems that capture key data. This report recommended collection of clinical care indicators, mental health, substance use, housing indicators, food insecurity, demographic and other social determinant of health data (i.e., income, reimbursement for medical services, etc.).
**Ethical considerations**			
Surveillance system development	5. Design surveillance system and associated infrastructure considering ethical principles including equity and accessibility (n=5).	Aiello (2020) ( ( ( ((44))))), Coltart (2018) ( ( ( ((45))))), Molldrem (2020) ( ( ( ((46))))), Moran-Gilad (2017) (47), Samuel (2018) ( ( ( ((48)))))	Aiello (2020) ( ( ( ((44))))): Outlines principles related to public health surveillance systems involving the internet and social media. Public health surveillance systems must be designed considering the following ethical principles: non-maleficence, beneficence, respect for autonomy, equity and efficiency. Coltart (2018) ( ( ( ((45))))): A review article that examined ethical issues in global HIV phylogenetic research. Risk-benefit assessments, with a special focus on key populations likely to be identified, should be conducted prior to designing, conducting and reporting phylogenetic studies. The rights and interests of individuals participating in these studies and the populations they represent need to be protected with clinically relevant results returned as soon as possible. Awareness regarding the social, legal, human rights and other aspects of society are needed with special focus on the effect of this research on criminalization and violence toward impacted populations (i.e., key populations). Risk mitigation strategies must be implemented to ensure that data cannot be re-linked for harmful purposes, training on privacy and confidentiality and including measures for ongoing monitoring and redress in cases of data misuse. True informed consent from participants is needed. Community engagement during the design, conduct and analysis of research is needed to ensure that projects are relevant to communities affected. Communication should be clear and understandable with special focus on sensitizing groups, such as communities, law enforcement and public health, to phylogenetic research and its implications. Equitable data sharing with governance structures to ensure accountability to communities is also required.
	6. Automate processes for data privacy and enhanced security with data anonymized, pseudonymized or using alternative identifiers in the event of data linkage (n=16).	Blais (2014) ( ( ( ((49))))), Boes (2020) ( ( ( ((50))))), Bublitz (2019) ( ( ( ((51))))), Campo (2020) ( ( ( ((52))))), Cauchi (2022) ( ( ( ((53))))), Fan (2010) ( ( ( ((54))))), Jeong (2022) ( ( ( ((22))))), Johnson (2018) ( ( ( ((55))))), McKerr (2015) ( ( ( ((56))))), Ndeikoundam Ngangro (2022) ( ( ( ((57))))), Polemis (2020) ( ( ( ((58))))), Rivière (2018) ( ( ( ((59))))), Sáez-López (2019) ( ( ( ((60), Severi (2023) ( ( ( ((61))))), Strobel (2019) (62), Trifirò (2014) ( ( ( ((63)))))	Boes (2020) ( ( ( ((50))))): Discusses the review of the national hepatitis B surveillance system in Germany. Physicians and laboratories report hepatitis B diagnoses to the local public health authorities, which report to state health authorities. Data is pseudonymized at reporting to state health authorities, which then submit data to the Robert Koch Institute. McKerr (2015) ( ( ( ((56))))): An evaluation of Taiwan’s national dengue surveillance system as part of the National Disease Surveillance System (NDSS). This system has an active component amongst travellers at ports of entry and a passive component amongst patients presenting for care at their healthcare providers, laboratory testing, notification to local health departments and subsequently the NDSS. Data is anonymized and extracted from the NDSS.
**Stakeholder consultation**			
Surveillance system operation	7. Community engagement and community-led monitoring throughout surveillance cycle (n=4).	Lee (2009) ( ( ( ((64))))), Sullivan (2022) ( ( ( ((14))))), Sweeney (2013) ( ( ( ((65))))), WHO (2021) ( ( ( ((35)))))	Lee (2009) (64): Commentary that proposes a US national privacy protection standard for public health data. Prior to the use or release of potentially identifiable data, affected community groups should informed and provided the opportunity to provide input in decision making processes. Sweeney (2013) ( ( ( ((65))))): Review of ethical considerations for using HIV surveillance data to facilitate HIV medical care. Prior to piloting a new program, community-based organizations were engaged in the development of strategies for the use and scale-up of HIV surveillance data.
	8. Consult with stakeholders on the development of specific information products with reporting in a sensitive/respectful manner that does not allow for the direct identification of affected individuals (n=8).	Burkom (2021) ( ( ( ((66))))), Cauchi (2022) ( ( ( ((53))))), Goldstick (2021) ( ( ( ((67))))), Hargrove (2018) ( ( ( ((68))))), Marshall (2017) ( ( ( ((69))))), Marzano (2023) ( ( ( ((70))))), Severi (2023) ( ( ( ((61))))), Simonsen (2016) ( ( ( ((71)))))	Marshall (2017) ( ( ( ((69))))): Examines the development of the Prevent Overdose Rhode Island (PORI) website. This website aimed to use public health surveillance data to provide information on drug overdoses to stakeholders and help direct those at risk to treatment. Prior to launching the web application, community and other stakeholders were sent email surveys. Over a hundred comments were received regarding how to improve the website. The next phase of evaluation will involve focus groups and user testing sessions to target content for audience. Marzano (2023) ( ( ( ((70))))): Reviews a real-time suicide surveillance system in Great Britain. Police forces submit forms with information on demographics, life events, mental health problems, previous police contact and circumstances surrounding death (i.e., time, date, location and method) to the British Transport Police. Involvement of police forces in the surveillance system has been controversial, due to previous history of criminalizing suicides. Further, reporting should be conducted in a respectful and anonymized manner.
	9. Provide training to public health stakeholders on the surveillance system (i.e., data collection) and knowledge mobilization initiatives to affected communities, public health professionals, clinicians and other stakeholders to improve public health surveillance of the condition of interest and demonstrate the public health value of the surveillance system (n=7).	Contoli (2016) ( ( ( ((72))))), Crain (2016) ( ( ( ((73))))), Hennenfent (2017) ( ( ( ((74))))), Magee (2011) ( ( ( ((75))))), Paul (2019) ( ( ( ((76))))), Rivière (2018) ( ( ( ((59))))), Smith (2011) ( ( ( ((77)))))	Contoli (2016) ( ( ( ((72))))): Discusses the implementation of PASSI d’Argento in Italy, a behavioural surveillance system monitoring health related behaviours in senior citizens. This surveillance system includes a web-based community of practice that offers workshops, communication tools and a forum for general discussions among stakeholders. Magee (2011) ( ( ( ((75))))): Describes the creation of a training program for the National Tuberculosis Surveillance System, which receives data from all 50 states, the District of Columbia, New York City, Puerto Rico and other US jurisdictions in the Pacific and the Caribbean. The US CDC developed a training course for the National Tuberculosis Surveillance System. This course had a self study and a facilitator led version. Training materials included visual, auditory, reading/writing and movement aspects with self-study modules to be used in the completion of the training course and to serve as reference material to take back to the participants’ jobs. Study questions and case studies were included, along with notes, comments and diagrams. Field testing with quantitative and qualitative data from field tests with experts were analyzed and pre-test and post-tests were designed and conducted. Trainers were also trained using the Teach-Back system. As the training program was successful, this was used as a model for similar trainings in other surveillance systems and jurisdictions.
	10. Provide a media toolkit and other knowledge translation tools for community-led social and behavioural change communication (n=1).	Sullivan (2022) ( ( ( ((14)))))	Sullivan (2022) ( ( ( ((14))))): Describes the data visualization tool America’s HIV Epidemic Analysis Dashboard (AHEAD). A toolkit for various users (i.e., jurisdictions, community-based organizations, advocacy organizations and federal agencies) was also available.
	11. Consult stakeholders during the development of and ongoing operation of surveillance system infrastructure and implement any feasible changes suggested (n=5).	Dubiniecki (2022) ( ( ( ((78))))), Marshall (2017) ( ( ( ((69))))), Toutant (2011) ( ( ( ((79))))), Yang (2020) ( ( ( ((80))))), Zhang (2017) ( ( ( ((81)))))	Dubiniecki (2022) ( ( ( ((78))))): Discusses the development and implementation of the Canadian Armed Forces Surveillance and Outbreak Management System (CAF SOMS). This system was developed by the National Contact Tracing Team and the Health Informatics Team and included the use of four distinct forms: case details, contagion elicitation, contact notification and contact follow-up. The database was presented to members of the National Contact Tracing Team for review. Perceptions of the system were positive and certain suggestions were acted on immediately. Other feasible suggestions that could not implemented immediately were prioritized for future implementation. Implemented changes received positive feedback. Toutant (2011) ( ( ( ((79))))): Examines the development of SUPREME, a web application to examine the effect of extreme meteorological events on public health. This web application assists public health surveillance by combining data from various datasets including, health, meteorological, demographic, air quality and geospatial data. The article explains how feedback from stakeholder surveys and meetings were used to identify information needs, provincial capabilities and develop specifications for the web application. Based on the given specifications, the web application, SUPREME, was developed.

Abbreviations: AHEAD, America’s HIV Epidemic Analysis Dashboard; CAF SOMS, Canadian Armed Forces Surveillance and Outbreak Management System; CDC, Centers for Disease Control and Prevention; HOSTED, Household Transmission Evaluation Database; NDSS, National Disease Surveillance System; PORI, Prevent Overdose Rhode Island; US, United States; WHO, World Health Organization

Novel findings regarding surveillance system and methodology addressed real- or near real-time data collection with surveillance system linkage and use of web-based applications in data collection. Web applications have been used in data submission to central agencies (15) and to receive data ( ( ( ((16))))). Timeliness of data has been improved through real-time submissions to central agencies by jurisdictions ( ( ( ((9))))) and institutions ( ( ( ((13))))).

Findings for data collection indicate the need to supplement information from passive surveillance systems with other data sources and collect data on demographic traits, disease staging (e.g., CD4) and the social determinants of health. Disease subtype from laboratory data can supplement routine surveillance ( ( ( ((17))))) and new systems developed by including population databases ( ( ( ((21))))). While data on age, gender, ethnicity, residence, exposure and place of infection is routinely collected for HIV ( ( ( ((38))))), clinical care indicators and social determinants of health data are needed (39).

Findings on ethical considerations included ethical, equitable and accessible surveillance system design and automated processes for data privacy alongside the ethical principles of non-maleficence, beneficence, respect for autonomy, equity and efficiency ( ( ( ((44))))). Risk-benefit assessments, awareness, risk mitigation, community engagement and equitable data sharing ( ( ( ((45))))) are needed in surveillance system design. Automated processes including pseudonymization ( ( ( ((50))))) at data submission and anonymization at data extraction ( ( ( ((56))))) enhance data privacy.

Stakeholder engagement is encouraged throughout the surveillance cycle and surveillance information product development. Training, knowledge mobilization and demonstration of the public health value of surveillance data, use of various knowledge translation tools and implementing changes recommended by stakeholders to the extent feasible are significant parts of surveillance system operation. Implementation included community engagement in decision and strategy making (64,65), as well as surveillance information product development ( ( ( ((69,70))))); provision of resources, workshops ( ( ( ((72))))) and training courses (75); audience specific knowledge translation toolkits ( ( ( ((14))))); and implementation of community suggested changes in the development of technical tools ( ( ( ((78,79))))).

### Long standing public health practices

Long standing public health practices were identified applying to surveillance system structure and methodology, data collection, ethical considerations and stakeholder consultation. (**Table 2**). Most practices were supported by multiple articles with additional details found in Table 2.

**Table 2:** Long standing practices in public health by theme and surveillance system stage

Surveillance system stage where relevant	Practice	Articles that support practices	Examples of practices
**Surveillance system structure and methodology**			
Surveillance system development	1. Delineate clear roles and responsibilities (n=3).	Gulaid (2012) ( ( ( ((82))))), Kodan (2021) ( ( ( ((83))))), O’Brien (2012) ( ( ( ((84)))))	Kodan (2021) ( ( ( ((83))))): Describes the process of implementing a maternal death surveillance system in Suriname as experienced by healthcare providers, committee members and public health experts. A working group oversaw the clear delineation of roles and responsibilities by specifying tasks. O’Brien (2012) ( ( ( ((84))))): Compares surveillance of transfusion-transmissible infections across the blood donation systems of five high income countries. Canada’s and France’s surveillance systems had differentiated responsibilities across programs and surveillance systems.
	2. Pilot test new data collection applications or systems in collaboration with other programs during surveillance system development (n=4).	Brady (2020) ( ( ( ((85))))), Karp (2017) ( ( ( ((86))))), Mao (2010) ( ( ( ((9))))), Sweeney (2013) ( ( ( ((65)))))	Brady (2020) ( ( ( ((85))))): Discusses the pilot testing of a voluntary community-based testing surveillance system for HIV in Ireland, with testing data submitted by clinics to the Health Protection Surveillance Centre. A steering group, including government and non-government organizations, was developed to oversee the development of the system including the development of a minimum dataset. Organizations were surveyed to develop database protocols and data collection tools. Karp (2017) ( ( ( ((86))))): Examines the National Antimicrobial Resistance Monitoring System (NARMS) in the US. This surveillance system receives data on retail meat from the US Food and Drug Administration (FDA) and food animals from the United States Department of Agriculture (USDA) in addition to data on human illness from the US Centers for Disease Control and Prevention (which receives data from state and local public health units). Data from the surveillance system is used as part of risk assessment in drug approvals the creation of risk management strategies using data on resistance trends, types and pathogen prevalence. Pilot studies were conducted on farms to assess data and sample collection processes and lessons learned were used in the development of antimicrobial resistance in food animal surveillance programs.
Surveillance system operation	3. Centralize and standardize data collection with standardized questionnaires and forms (n=7).	Kebisek (2021) ( ( ( ((87))))), Lee (2009) ( ( ( ((64))))), Mao (2010) ( ( ( ((9))))), Mohammed (2016) ( ( ( ((88))))), O’Brien (2012) ( ( ( ((84))))), Weston (2018) ( ( ( ((34))))), Zhang (2012) ( ( ( ((89)))))	Kebisek (2021) ( ( ( ((87))))): Describes the process of conducting near real-time COVID-19 surveillance among the US Army population during the first year of the pandemic through the integration of several Department of Defense surveillance systems. Mao (2010) ( ( ( ((9))))): Describes the creation of the Chinese national, web-based HIV/AIDS information system. Data collection on HIV testing, prevention and treatment is standardized across provinces.
Surveillance system evaluation	4. Conduct periodic evaluations of surveillance system (n=5).	Cohen (2014) ( ( ( ((15))))), Lee (2009) ( ( ( ((64))))), Petty-Saphon (2019) ( ( ( ((90))))), Rossi (2017) ( ( ( ((41))))), Trepka (2016) ( ( ( ((91)))))	Cohen (2014) ( ( ( ((15))))): Reviews the status of the US National HIV Surveillance System in 2013. The surveillance system will continue to evolve in response to the need for high-quality data and the findings from this evaluation. Rossi (2017) ( ( ( ((41))))): Reviews the factors that influence the accuracy of infectious disease monitoring in migrants in Europe. The improvement of existing surveillance systems was recommended after this review.
**Data collection**			
Surveillance system operation	5. Resolve duplicates and data quality issues prior to upload into database (n=3).	Chapman (2011) ( ( ( ((19))))), Jung (2018) ( ( ( ((24))))), Strobel (2019) ( ( ( ((62)))))	Chapman (2011) (19): Examines the development of the Virginia Vital Events and Screening Tracking System (VVESTS), which is created by merging the Virginia Congenital Anomalies Reporting and Education System (VaCARES) (passive surveillance system) and the Virginia Early Hearing Detection and Intervention Program (VEHDIP). As duplicate records are a challenge, these records are assessed by querying for combination of variables. Strobel (2019) ( ( ( ((62))))): Examines the evaluation of the Intellectual Disability Exploring Answers (IDEA) surveillance system in Western Australia, which was created by merging data from the Department of Education Western Australia and the Disability Services Commission (DSC). The surveillance system can be linked to administrative data as necessary. Data quality is assessed by cross-checking data across different systems and conducting cross-tabulations as needed.
	6. Manually verify data linkages (n=2).	Chapman (2011) (19), Leal (2010) ( ( ( ((27)))))	Chapman (2011) ( ( ( ((19))))): Examines the development of the VVESTS, which is created by merging the VaCARES (passive surveillance system) and the VEHDIP. Data linkages are manually reviewed for accuracy. Leal (2010) ( ( ( ((27))))): Examines the creation of a surveillance system for bloodstream infection in Calgary, which was created by merging regional laboratory data with data from hospital administrative databases. Regional laboratory data was merged with hospital administrative databases. Medical records were manually reviewed to verify data linkages.
**Ethical considerations**			
Surveillance system development	7. Review relevant legislations specific to the condition under surveillance (i.e., criminalization), their implications, privacy policies/legislations, other relevant legislations and obtaining the needed approvals to design surveillance systems in compliance with these laws and policies (n=13).	Abecasis (2018) ( ( ( ((92))))), Blais (2014) ( ( ( ((49))))), Bublitz (2019) ( ( ( ((51))))), Campo (2020) ( ( ( ((52))))), Condell (2016) ( ( ( ((93))))), Fritsch (2019) ( ( ( ((94))))), Ghosh (2012) ( ( ( ((8))))), Hoppe (2022) ( ( ( ((95))))), Leitner (2018) ( ( ( ((96))))), Marzano (2023) ( ( ( ((70))))), Ndeikoundam Ngangro (2022) ( ( ( ((57))))), Scaduto (2010) ( ( ( ((97))))), Trifirò (2014) (63)	Blais (2014) ( ( ( ((49))))): Discusses the evaluation of the Québec Integrated Chronic Disease Surveillance System (QICDSS). This surveillance system was created by linking several databases: the health insurance registry, hospitalization database, vital statistics death database, physician claims database and pharmaceutical services databases. The QICDSS creation process was evaluated by government bodies with legal ownership of databases, the public health ethics committee and *Commission d’accès à l’information du Québec*. Ghosh (2012) ( ( ( ((8))))): Reviews the National Tuberculosis Surveillance System in the US, created by linking epidemiological data submitted by state and territorial public health departments with laboratory data from the National Tuberculosis Genotyping Service. Compliant with federal legislation, identifying information is not stored and user credentialing is also maintained.
Surveillance system operation	8. Create data sharing and use agreements with surveillance partners with variables and additional sources limited to purposes of surveillance system for privacy reasons (n=3).	Blais (2014) ( ( ( ((49))))), Ghosh (2012) ( ( ( ((8))))), Strobel (2019) ( ( ( ((62)))))	Blais (2014) ( ( ( ((49))))): Discusses the evaluation of the QICDSS. This surveillance system was created by linking several databases: the health insurance registry, hospitalization database, vital statistics death database, physician claims database and pharmaceutical services databases. Only relevant variables were used in the creation of the QICDSS. Strobel (2019) ( ( ( ((62))))): Examines the evaluation of the IDEA surveillance system in Western Australia, which was created by merging data from the Department of Education Western Australia and the DSC. Variable collection is limited for privacy reasons. Data sharing agreements governing data provision from data sources are present.
	9. Restrict data access and sharing on need-to-know basis for data integrity and privacy with access monitored using logs (n=4).	Blais (2014) ( ( ( ((49))))), Ghosh (2012) ( ( ( ((8))))), Marzano (2023) ( ( ( ((70))))), Ndeikoundam Ngangro (2022) ( ( ( ((57)))))	Blais (2014) ( ( ( ((49))))): Discusses the evaluation of the QICDSS. This surveillance system was created by linking several databases: the health insurance registry, hospitalization database, vital statistics death database, physician claims database and pharmaceutical services databases. Data access is logged and limited to the team working within the surveillance system. Ndeikoundam Ngangro (2022) ( ( ( ((57))))): Discusses the feasibility of an automated surveillance system for sexually transmitted infections in France. Sexually transmitted infections clinics submit data to *Santé Publique France* through a web portal. Data access is limited to authorized personnel.
	10. Seek ethics approvals for specific projects outside of routine surveillance (i.e., manuscripts) (n=2).	Fan (2010) ( ( ( ((54))))), Sáez-López (2019) ( ( ( ((60)))))	Fan (2010) ( ( ( ((54))))): Describes the development of the Alberta Real Time Syndromic Surveillance Network (ARTSSN), a real-time surveillance system obtaining data from several different databases simultaneously: Health Link calls, emergency department visits, school absenteeism, laboratory reports and online forms. Research uses of the data must be approved with the process simplified due to the pseudonymization of data. Sáez-López (2019) ( ( ( ((60))))): Discusses the evaluation of Portuguese influenza surveillance system, which has a sentinel and non-sentinel component. The non-sentinel component operates by having laboratories associated with the Portuguese Laboratory Network for the Diagnosis of influenza infection. Approval for specific projects is granted by the Health Ethic Committee of National Institute of Health Doutor Ricardo Jorge.
	11. Obtain informed consent from individuals consulted as part of surveillance system evaluation (n=3).	Johnson (2018) ( ( ( ((55))))), McKerr (2015) ( ( ( ((56))))), Rivière (2018) ( ( ( ((59)))))	McKerr (2015) ( ( ( ((56))))): This article is an evaluation of Taiwan’s national dengue surveillance system as part of the National Disease Surveillance System (NDSS). This system has an active component amongst travellers at ports of entry and a passive component amongst patients presenting for care at their healthcare providers, laboratory testing, notification to local health departments and subsequently the NDSS. Verbal informed consent was obtained from those interviewed. Rivière (2018) ( ( ( ((59))))): Summarizes an evaluation of the surveillance of bovine tuberculosis in France. Passive surveillance is conducted on specimens killed by hunters and dead or dying animals with data submitted to local veterinary services and the National Hunting and Wildlife Office. There is also an active surveillance component on living specimens with data submitted to the local veterinary services. Verbal informed consent was obtained prior to interviews.
**Stakeholder consultation**			
Surveillance system development	12. Build stakeholder relationships with each other and with surveillance system based on ongoing communication with stakeholders using tools such as communication plans including multiple methods of communication (n=16).	Asburry (2019) ((98)), Burkom (2021) ( ( ( ((66))))), Cauchi (2022) ( ( ( ((53))))), Contoli (2016) ( ( ( ((72))))), Crain (2016) ( ( ( ((73))))), Dawson (2016) ( ( ( ((99))))), European Food Safety Authority (2023) ( ( ( ((100))))), Huot (2019) ( ( ( ((101))))), Johnson (2018) ( ( ( ((55))))), McGill (2023) (10), Rivière (2018) ( ( ( ((59))))), Schönfeld (2018) ( ( ( ((102))))), Schwartz (2022) ( ( ( ((103))))), Smith (2011) ( ( ( ((77))))), Strobel (2019) (62), Takla (2012) ( ( ( ((104)))))	Smith (2011) ( ( ( ((77))))): Examines the implementation of a surveillance system model for child maltreatment developed by the US CDC. Numerous data sources are used, such as: death certificates, homicide files, medical examiner records, child protective services records, child welfare registries and Child Death Review Team (CDRT) reports. Respondents indicated that personal collaborative relationships between stakeholders were essential. However, frequent staff turn over and low meeting attendance were identified as hindrances in developing these relationships. Schwartz (2022) ( ( ( ((103))))): Outlines the development of a COVID19 surveillance system in Memphis, US. The surveillance system combined regional laboratory COVID19 testing and epidemiological data with electronic medical records, social determinant and environmental data. The surveillance system included standard operating procedures for team meetings and community outreach in addition to a formal community outreach leadership team.
Surveillance system operation	13. Ensure that the surveillance system is sustainable, ongoing and well resourced with the ability support the participation of all stakeholders in the surveillance system (n=15).	Asburry (2019) (98), Crain (2016) ( ( ( ((73))))), Dawson (2016) (99), Ehlman (2021) ( ( ( ((105))))), Fedorowicz (2010) ( ( ( ((106))))), Hennenfent (2017) ( ( ( ((74))))), Jermacane (2019) ( ( ( ((107))))), Johnson (2018) ( ( ( ((55))))), McGill (2023) ( ( ( ((10))))), Moore (2012) ( ( ( ((11))))), Paul (2019) ( ( ( ((76))))), Schönfeld (2018) ( ( ( ((102))))), Smith (2011) ( ( ( ((77))))), Wijayasri (2016) ( ( ( ((36))))), Wong (2022) ( ( ( ((108)))))	Ehlman (2021) ( ( ( ((105))))): Discusses the evaluation of the National Electronic Injury Surveillance System. Coders in emergency departments complete data entry in the system, sending data to the Consumer Product Safety Commission, which submits data to the US CDC. Issues identified with the surveillance system include multiple approvals, updates and training required when the system is updated, data completion, issues with laptops and staff turnovers and the need to replace sites if any drop out of surveillance system. Moore (2012) ( ( ( ((11))))): Describes the evaluation of the US Department of Defense Serum Repository and Defense Medical Surveillance System, linking laboratory data with demographic data to create a surveillance system for the health of the military. Recommendations included: setting and communicating priorities for resources, strengthening and resourcing organizational oversight, distinct chain of command for receiving guidance and resource from policymakers in addition to adequate staffing to meet user needs.
Surveillance system evaluation	14. Stratify consultations based on different groups and include the following in surveillance system evaluation: surveys, focus groups and/or meetings and interviews (n=15).	Ehlman (2021) ( ( ( ((105))))), Fedorowicz (2010) ( ( ( ((106))))), Halliday (2013) ( ( ( ((109))))), Hennenfent (2017) (74), Jermacane (2019) ( ( ( ((107))))), Johnson (2018) ( ( ( ((55))))), Kunze (2022) ( ( ( ((110))))), Paul (2019) (76), Pratt (2020) ( ( ( ((111))))), Rivière (2018) ( ( ( ((59))))), Schönfeld (2018) ( ( ( ((102))))), Smith (2011) ( ( ( ((77))))), Takla (2012) ( ( ( ((104))))), Yang (2020) ( ( ( ((80))))), Zhang (2017) ( ( ( ((81)))))	Halliday (2013) (109): Describes the surveillance system evaluation of the Australasian Maternity Outcomes Surveillance System. In this surveillance system, monthly data requests are emailed to maternity unit data collectors who can either submit data or submit a nil report through web-based reporting. Data collected would then be accessed by study investigators for research and dissemination. In addition to conducting an anonymous online survey of stakeholders, documentation from advisory and project meetings in addition to official correspondence were reviewed to identify previous concerns, questions, comments and feedback. Takla (2012) (104): Discusses an event-specific surveillance system developed for the FIFA women’s world cup in Germany in 2011. The local district where the world cup was held would report to the Robert Koch Institute (national health authority) through state health authorities, with infectious disease notifications being provided daily. The national health authority would provide feedback and summary reports to the ministry of health, district and state health authorities twice weekly in addition to phone conferences with key stakeholders as needed. Results from the evaluation were stratified across district vs. state health authorities.

Abbreviations: ARTSSN, Alberta Real Time Syndromic Surveillance Network; CDC, Centers for Disease Control and Prevention; CDRT, Child Death Review Team; DSC, Disability Services Commission; FDA, Food and Drug Administration; IDEA, Intellectual Disability Exploring Answers; NARMS, National Antimicrobial Resistance Monitoring System; NDSS, National Disease Surveillance System; QICDSS, Québec Integrated Chronic Disease Surveillance System; US, United States; USDA, United States Department of Agriculture; VaCARES, Virginia Congenital Anomalies Reporting and Education System; VEHDIP, Virginia Early Hearing Detection and Intervention Program; VVESTS, Virginia Vital Events and Screening Tracking System

Surveillance system structure and methodology should include clearly defined roles and responsibilities and pilot testing during surveillance system development. Questionnaires were centralized and standardized during operation. Periodic evaluation was also conducted. Measures to enhance surveillance system structure included: working groups specifying tasks ( ( ( ((83))))) and program-specific responsibilities (84), pilot testing of surveillance systems in data collection settings ( ( ( ((85,86))))), integration (87) and standardization across jurisdictions ( ( ( ((9))))) and periodic evaluations to understand factors affecting accuracy ( ( ( ((41))))) and ensure the relevance of these factors, as high-quality data is needed ( ( ( ((15))))).

Data collection during operation should include resolving duplicates and data quality issues prior to upload onto databases and manually verifying data linkages. Structured queries (19,64) and cross-tabulations (53,62) and manual verification between data sources (19,64) or against raw data, such as medical records ( ( ( ((27))))), also ensure high data quality.

Ethical considerations include legislative and policy review and approval during development. Data sharing agreements, data access monitoring with need-to-know restriction and non-routine project-specific ethics approval were present during ongoing operation. Informed consent from stakeholders was obtained during evaluation. Ethics committee evaluation ( ( ( ((49))))), data protection for legislative compliance ( ( ( ((8))))), minimum data collection for privacy ( ( ( ((49))))), data sharing agreements governing data provision (62), maintaining data access logs ( ( ( ((49))))) with data access limited to authorized individuals (57), simplified oversight processes for extra projects ( ( ( ((60))))) accounting for existing data protection ( ( ( ((54))))) and verbal informed consent during evaluation interviews ( ( ( ((56,59))))) all ensure ethical public health surveillance.

During surveillance system development, inter- and intra-stakeholder relationships should be built and facilitated using various tools. Dedicated resources should be provided for ongoing operation. Consultations during periodic evaluations should be stratified by stakeholder group. Consistent staff (77), formal outreach processes ( ( ( ((103))))), dedicated resources ( ( ( ((105))))), senior guidance and oversight (11) and consultations stratified by jurisdictional level during evaluation ( ( ( ((104))))) supplemented by existing communications ( ( ( ((109))))) improves stakeholder engagement.

## Discussion

Several proposed approaches with respect to areas for improvement, such as surveillance system infrastructure and methodology, data collection, ethical public health surveillance and stakeholder engagement were identified through this literature review. As the use of evidence-informed policy and programs is a guiding principle of the *Government of Canada’s sexually transmitted and blood-borne infections (STBBI) action plan 2024–2030*, surveillance data has been emphasized as a guide for developing and implementing programs and interventions ( ( ( ((2,3))))). Thus, it is important to periodically review and modernize surveillance systems to ensure their effectiveness. Key attributes of surveillance systems have been defined as follows by the United States Centers for Disease Control and Prevention (CDC), as part of its guidelines on public health surveillance system evaluation to develop effective surveillance systems: simplicity, flexibility, data quality, acceptability, sensitivity, predictive value positive, representativeness, timeliness and stability ( ( ( ((112))))). Findings from the current review show that effective surveillance systems are characterized as standardized across jurisdictions, timely, developed and tested in conjunction with stakeholders and linked to other data sources. Data collection may benefit from including demographic information, disease staging and social determinant of health indicators, supplemented by other data sources as needed and include data quality and duplicate checks. Ethical surveillance system design considers equity and accessibility, privacy issues, legislation and policy review and informed consent. Effective stakeholder engagement is meaningful and conducted at all stages of public health surveillance, integrating various methods and programs.

The positive impact of modernizing national HIV surveillance systems was noted previously in the United States, since it updated its National HIV Surveillance System in 2013 with enhancements, such as improvements in data collection on sexual orientation, gender identity, social determinants of health and improved data deduplication procedures to enhance data quality ( ( ( ((113))))). This update has resulted in an increased capacity to rapidly respond to HIV clusters and support ongoing viral suppression through “data-to-care” activities ( ( ( ((113))))). Although the National HIV Surveillance Program in Canada presents information on the epidemiology of HIV on a national level, the program does not have a centralized database with consistent data standards and relies on data received from thirteen PTs. This affects the timeliness of surveillance data, impacting the relevance of data used to inform evidence-based policies and programs for HIV prevention and treatment. As the data for the Canadian National HIV Surveillance Program is provided by the PTs, data linkages to additional data sources for other social determinants of health data is not available. As such, proposed approaches included incorporating a centralized database, data quality procedures and additional data sources and social determinant of health data collection as part of the National HIV Surveillance Program renewal. Furthermore, it is important to update the National HIV Surveillance Program to better address the burden of HIV in Canada. It is anticipated that the potential renewal of the program in Canada will result in improved data collection and quality, providing key evidence needed to meet the federal government’s commitments under its STBBI action plan ( ( ( ((2,3))))), improved public health outcomes and improved outcomes for those diagnosed and living with HIV.

The proposed improvements to surveillance system infrastructure and methodology may improve the simplicity, timeliness and stability of the National HIV Surveillance Program as data standardization across jurisdictions simplifies data submission, which in turn could improve timeliness and stabilizes data submission processes. Proposed approaches for ethical surveillance system design may result in improved ethical surveillance practices and subsequently increase the acceptability of the surveillance system. Improving stakeholder engagement using the proposed approaches could improve the acceptability as stakeholders may be more likely to engage with the surveillance program.

Although numerous proposed approaches addressed various areas of improvement, not all findings from this literature review may be applicable to national HIV surveillance in Canada. For instance, a real-time HIV surveillance system ( ( ( ((35))))) may not be needed at the national level, as provincial, territorial and local public health units would be primarily addressing HIV outbreaks or clusters, as opposed to the National HIV Surveillance Program and the national program serves to provide a national overview of HIV epidemiology, instead of localized data.

Findings from this review were consistent with findings from consultations with data providers and community organizations conducted as part of the National HIV Surveillance Program’s review and renewal process and the recommendations in the *Pan-Canadian Health Data Strategy: Toward a world-class health data system* ( ( ( ((114))))). While the need for meaningful community engagement and involvement has been acknowledged previously (115,116), the program’s consultations and the Pan-Canadian Health Data Strategy (PCHDS) (114) emphasized the importance of community consultations regarding data collection, interpretation and storage. Regarding data infrastructure and quality, the program’s consultations and the PCHDS ( ( ( ((114))))) suggest developing consistent data standards, with the PCHDS advocating for timely data access, health data literacy promotion and data-driven social and technological innovation ( ( ( ((114))))). Furthermore, the program’s consultations recommended the collection of information on sex, gender, sexual orientation, race and/or ethnicity and disease staging (e.g., CD4 cell count). The PCHDS proposed prioritizing the following related to data governance: ethical data use, First Nations, Inuit and Metis data sovereignty, oversight, federal leadership and measures to address security and privacy concerns ( ( ( ((114))))). The findings of this review are consistent with the Public Health Agency of Canada’s vision for what public health surveillance should look like by 2030, with enhanced surveillance workflows, high-quality data, improved data sharing and linkage, community partnerships and public engagement ( ( ( ((117))))). The National HIV Surveillance Program’s ongoing collaboration with data providers and community members to update data management, improve data collection on key variables and stakeholder engagement represents meaningful efforts to advance HIV surveillance in Canada.

This literature review provides a comprehensive overview of current and future proposed approaches in public health surveillance relevant to Canada’s national HIV surveillance. It contains evidence gathered from diverse sources over a broad timeframe, encompassing technological advances and surveillance system enhancements in response to public health events. As the epidemiology of diseases change over time, surveillance systems are updated to ensure relevance, resulting in the development and incorporation of new public health practices. The wide timeframe allows for the aggregation of practices gained from experience dealing with various public health events (e.g., COVID-19 pandemic) and technological updates, such as databases incorporating automated data linkage. Including information from a wide range of jurisdictions, with similar surveillance systems or public health contexts, allowed for a wider range of public health practices to be incorporated in this literature review for future discussion. In addition, the inclusion of information on other surveillance systems, instead of solely focusing on HIV surveillance systems, allowed for the inclusion of various practices. These include linked national and state tuberculosis surveillance systems in the United States ( ( ( ((8))))) and additional methods for stakeholder collaboration and communication through resources tailored to stakeholders in a surveillance system examining health related behaviours in senior citizens in Italy ( ( ( ((72))))). With the potential incorporation of these practices, linking the National HIV Surveillance Program to other surveillance systems or data sources could improve data collection on HIV and other relevant indicators. Resources tailored to stakeholders could improve the relevance of surveillance information products such as surveillance reports and infographics, as well as improve stakeholder engagement with the national program. Incorporating grey literature allowed novel and innovative practices found in emerging research to be included. Information can often be found in evidence reviews or reports in addition to published journal articles, such as a World Health Organization report on achieving key goals related to HIV, hepatitis and sexually transmitted infections, outlining various advances, such as case-based surveillance systems enhanced with additional data for improved care (35).

### Limitations

This review was limited to information available online (i.e., additional information may be found in internal surveillance protocols and standard operating procedures). There may be additional technical details from these internal documents that may guide the development and operation of data management systems and other surveillance system tools that may not be accessible for review. While attempts were made to include articles primarily focused on passive surveillance systems, decisions regarding inclusion were sometimes difficult. For instance, articles regarding more recent surveillance systems often included components involving active or sentinel surveillance that supplemented passive surveillance. Additionally, this review was not a systematic review. Therefore, quality of evidence and risk of bias were not assessed. As quality of evidence was not considered and risk of bias was not assessed, evidence presented here could not be weighted based on these factors, rendering all sources as equal. This allows for over emphasis on lower quality and biased sources and under emphasis on higher quality sources with less bias. Despite these limitations, the results of this review provide evidence for improving surveillance practices as the National HIV Surveillance Program seeks to modernize surveillance practices and processes.

In addition to identifying possible directions for improvement of Canada’s national HIV surveillance program, this literature review summarizes public health surveillance practices for HIV and other conditions. The potential for applying public health surveillance practices for other conditions to HIV surveillance has been highlighted in this review and it serves as a valuable reference compiling information on proposed approaches to improving surveillance system infrastructure and methodology, data collection, ethical public health surveillance and stakeholder engagement in a single place. As such, this review may be relevant to other surveillance systems looking to review and renew their own systems.

## Conclusion

In conclusion, as part of a comprehensive review of Canada’s HIV surveillance program, several current and future proposed approaches for surveillance systems were identified. Promising potential innovations include updates to data collection infrastructure, inclusion of new data elements (e.g., CD4 cell count), improving collection of existing data elements (i.e., sex and gender, race and/or ethnicity, HIV exposure information), developing a data governance framework and ongoing engagement with data providers and communities. Next steps include examining ways to validate and operationalize these key findings in collaboration with data providers and affected communities.

## Appendix

**Table A1:** Articles included that did not meet one or more inclusion criteria

Article	Inclusion criteria not met	Rationale for inclusion
Kodan (2021) ( ( ( ((83)))))	Study location in: North America, Australia, New Zealand, United Kingdom, Western Europe, Europe, Japan, Singapore, South Korea, Taiwan, Malaysia This study was conducted in Suriname.	This article was included as it outlined the development and operation of a working group overseeing the assignment of tasks and clearly delineating roles and responsibilities. This is relevant during stakeholder collaboration during a potential renewal.
Mao (2010) ( ( ( ((9)))))	Study location in: North America, Australia, New Zealand, United Kingdom, Western Europe, Europe, Japan, Singapore, South Korea, Taiwan, Malaysia This study was conducted in China.	This article was directly related to HIV surveillance and detailed the development of a national web-based HIV/AIDS information system based on real-time data. This is relevant to surveillance structure methodology and infrastructure during a potential renewal.
Zhang (2012) ( ( ( ((89)))))	Study location in: North America, Australia, New Zealand, United Kingdom, Western Europe, Europe, Japan, Singapore, South Korea, Taiwan, Malaysia This study was conducted in China.	This article had discussed the effect of social stigma and political structures on HIV surveillance in China and highlighted the importance of centralized data collection. This is relevant to data collection during a potential renewal.
Lee (2009) ( ( ( ((64)))))	Published on or after January 1, 2010 This article was published in 2009.	This article was a commentary in the United States proposing a national privacy protection standard for public health data and emphasized the importance of community groups providing input in decision making, standardized data collection and periodic surveillance system evaluation. This is relevant during stakeholder collaboration during a potential renewal.

Additional supplementary information on search strategies and included articles is available upon request. Please contact hass@phac-aspc.gc.ca
